# Type-2 diabetes alters the basal phenotype of human macrophages and diminishes their capacity to respond, internalise, and control *Mycobacterium tuberculosis*

**DOI:** 10.1590/0074-02760170326

**Published:** 2018-02-19

**Authors:** Nallely Lopez-Lopez, Ana Gabriela Ramos Martinez, Mariana Haydee Garcia-Hernandez, Rogelio Hernandez-Pando, Julio Enrique Castañeda-Delgado, Geanncarlo Lugo-Villarino, Céline Cougoule, Olivier Neyrolles, Bruno Rivas-Santiago, Monica Alejandra Valtierra-Alvarado, Marisela Rubio-Caceres, Jose Antonio Enciso-Moreno, Carmen Judith Serrano

**Affiliations:** 1 Instituto Mexicano del Seguro Social, Unidad de Investigación Biomédica Zacatecas, Zacatecas, Mexico; 2 Universidad Autónoma de San Luis Potosí, Escuela de Medicina, Departamento de Inmunología, San Luis Potosí, Mexico; 3 Instituto Nacional de Ciencias Médicas y de la Nutrición Salvador Zubirán, Departamento de Patología, Sección de Patología Experimental, Ciudad de México, México; 4 Consejo Nacional de Ciencia Y Tecnología-CONACyT, Cátedras CONACyT, Unidad de Investigación Biomédica Zacatecas, Instituto Mexicano del Seguro Social, Zacatecas, México; 5 Université Paul Sabatier, Centre National de la Recherche Scientifique, Institut de Pharmacologie et Biologie Structurale, Toulouse, France; 6 Instituto Mexicano del Seguro Social, Unidad de Medicina Familiar No. 4, Guadalupe, Zacatecas, México

**Keywords:** diabetes, tuberculosis, virulence, macrophages

## Abstract

**BACKGROUND:**

Type 2 diabetes (T2D) is a risk factor for the development of tuberculosis (TB), although the associated mechanisms are not known.

**OBJECTIVES:**

To study the association between T2D and the basal phenotype of macrophages, and their immune response to *Mycobacterium tuberculosis* (Mtb) infection.

**METHODS:**

We evaluated the influence of T2D on the response of monocyte-derived macrophages (MDM) to Mtb in patients with T2D (n = 10) compared to healthy subjects (n = 9), before and after infection with Mtb clinical isolates bearing different degrees of virulence. The levels of cell surface markers for activation secreted cytokines and chemokines, bacterial association, and intracellular bacterial growth were evaluated.

**FINDINGS:**

The expression levels of HLA-DR, CD80, and CD86 were low while those of of PD-L1 were high in uninfected MDMs derived from patients with diabetes; as a result of Mtb infection, changes were only observed in the expression levels of PD-L1. The levels of cytokines (*e.g.*, IL-6, IL-1β, IL-10, and IL-12) and chemokines (*e.g.*, MCP-1, MIG, and RANTES) are perturbed in MDMs derived from patients with diabetes, both before infection and in response to Mtb infection. In response to the more virulent Mtb strains, the levels of association and bacterial clearance were diminished in MDMs derived from patients with diabetes.

**CONCLUSIONS:**

T2D affects the basal activation state of the macrophages and its capacity to respond and control Mtb infection.

Tuberculosis (TB), whose etiological agent is *Mycobacterium tuberculosis* (Mtb), is an infectious disease that spreads from person to person through the air. According to a 2015 World Health Organization (WHO) report, while considerable progress has been made against TB in terms of controlling the mortality and incidence rates, it still claims around 1.8 million lives annually, making it one of the deadliest diseases caused by a single infectious agent. TB infection is more critical in people with compromised immune systems caused by well-known factors, such as age, malnutrition, and co-infection with human immunodeficiency virus (HIV), as well as in patients with less well-known risk factors, such as diabetes (WHO 2016).

Diabetes is a chronic and progressive disease that is characterised by high levels of blood glucose, leading to physical complications in the human body and, if not treated correctly, to premature death. Global socio-economic and demographic changes over the last half century have resulted in an unprecedented increase in diabetes. It is estimated that the number of people living with diabetes will rise from 382 million (in 2013) to 592 million by 2035. About 85-95% of the global prevalence of diabetes is attributed to Type-2 Diabetes (T2D), which results from the body’s failure to adequately respond to insulin.

Historically, the rise of T2D occurred in high-income settings, and it became known as a “disease of affluence”. Today, this first impression is no longer true, as approximately 80% of people with T2D currently live in low- and middle-income countries. With TB mainly affecting the poor, and estimated to be latent in one fourth of the world population (about 1.7 billion people), the geographical overlap with T2D represents a serious public health issue that needs to be addressed at all levels. Recent epidemiological studies have shown that T2D makes a person three times more likely to develop TB, and as a consequence, it is predicted that 15% of the TB burden world-wide is now associated with T2D (Lönnroth et al. 2014). In fact, similar to other conditions rendering an immunocompromised host, T2D is known to have an all-around effect on the natural life cycle of TB, including a higher risk of the patient becoming infected with Mtb (latent infection), a higher lifetime risk of TB reactivation, and a worsening of clinical features (Hodgson et al. 2015). As T2D spreads globally, it will cause more people to develop TB, and consequently drastically change the landscape of TB care and prevention; the WHO has recently identified T2D as neglected risk factor for re-emerging TB.

In Mexico, TB continues to represent a serious public health problem; as of 2015, the TB incidence rate was 21 per 100,000 inhabitants (WHO 2016). In terms of T2D incidence, Mexico has become one of the countries with the highest national prevalence, currently standing at over 11.8% of the population (IDF 2014). The convergence of both diseases in Mexico is also on the rise, and is considered to be more critical than the TB/HIV comorbidity. Together with India, it is thought that T2D can account for more than 30% of the TB cases in Mexico. To better understand and control the looming T2D/TB co-epidemic, fundamental research is currently focused on how T2D influences the host immune system and its control of Mtb. T2D is a multifactorial metabolic disease ultimately resulting in a “low-grade” chronic inflammation, and whose long-term effects have sparked interest in the role of T2D in modulating the macrophage (MF) compartment.

MFs are key to the aetiology of TB due to their dual role as a primary host cell reservoir for Mtb, as well as being effector cells that control and eliminate Mtb. Little is known about the state of MF activation during the low-grade chronic inflammation linked to T2D; much of what we know is derived from the context of obesity. It has been proposed that during weight gain, and the subsequent increase in low-grade chronic inflammation, MFs undergo a phenotypic switch from an anti-inflammatory phenotype towards a pro-inflammatory one, and are thus associated with the emergence of systemic insulin resistance (Lumeng et al. 2007). Despite existing evidence that human monocytes are altered in the context of T2D in terms of low phagocytosis/association rates and high chemotactic activity (Restrepo et al. 2014), there is no clear indication if MFs undergo these alterations and whether this influences immunity against Mtb.

In the present study, we present preliminary data suggesting that T2D influences the activation state of the human MF compartment in terms of their basal phenotype and inflammatory potential. Moreover, these MFs exhibit a diminished capacity to recognise, respond, and control the bacillus, in particular against the more virulent Mtb strains. Taken together, our findings point to the fact that one of the long-term effects enacted by low-grade chronic inflammation associated with T2D is an altered and deficient MF compartment, which therefore represents a cellular mechanism for susceptibility to TB.

## SUBJECTS AND METHODS

*Recruitment of participants* - The control subjects and patients with T2D were recruited from the Family Medical Unit No. 4 of the Mexican Institute of Social Security at Guadalupe, Zacatecas, Mexico. All participants were between 35 to 65 years of age. All participants were interviewed to rule out the possibility of active TB and present or past HIV infection. Those with a history of TB or showing a positive tuberculin skin test (TST) (Sanofi Pasteur, Canada) were excluded from the study. In addition, subjects receiving treatment with TNF-α blockers were excluded due to possible alterations in their immune response. The weight and height were recorded and were used to calculate body mass index (BMI). Peripheral blood was obtained to determine fasting plasma glucose, glycated haemoglobin (HbA1c), triglycerides, total cholesterol, and the lipid profile, including very low density lipoprotein (VLDL), low-density lipoprotein (LDL), and high density lipoprotein (HDL*)* levels*.* All patients with diabetes included in this study had HbA1c levels ≥ 8%, and had been diagnosed as being diabetic for at least 5 years. Diabetes was defined according to the 2015 American Diabetes Association (ADA) criteria that includes any of the following parameters: a HbA1c ≥ 6.5%, or a fasting plasma glucose ≥ 126 mg/dL, or a plasma glucose at 2 h after a glucose tolerance test (75 g glucose) of ≥ 200 mg/dL, or a plasma glucose of ≥ 200 mg/dL in patients with classic symptoms of hyperglycaemia, including polyuria, polydipsia, inexplicable weight loss, excessive hunger, fatigue, delay in wound closing, frequent skin infections. All patients with diabetes were receiving glucose- lowering drugs (glibenclamide or metformin). Subjects with no T2D were selected if they had HbA1c levels ≤ 6.5%, and met the inclusion criteria explained above for all participants. A sample of 50 mL of whole blood was obtained from patients with diabetes, or from individuals who were not patients with diabetes, while they were visiting the hospital for their monthly medical check-up. All procedures were reviewed and approved by the National Health Research and Ethics Committee of the Instituto Mexicano del Seguro Social (ID R-2103-785-048). All participants provided their written informed consent.

*Preparation of human monocytes and monocyte-derived macrophages (MDMs)* - Peripheral blood mononuclear cells (PBMCs) from healthy subjects and Patients with T2D were obtained by density gradient centrifugation using Ficoll-Paque PLUS (Ficoll-Paque PLUS (GE Healthcare, Little Chalfont, Buckinghamshire, UK). Monocytes were then purified using negative selection (Miltenyi, Auburn, CA, USA) and LD MACS magnetic columns (Miltenyi, USA). Monocytes were resuspended in RPMI-1640 medium (Gibco, Life Technologies, Grand Island, NY, USA) supplemented with 20% autologous serum, 1% penicillin-streptomycin (Gibco, Life Technologies) and 25 ng/mL GM-CSF (Miltenyi). Monocytes were seeded at a density of 1.5 × 10^5^ cells/well in 24-well plates (Corning-Costar, NY, USA) and grown for 7 days at 37ºC and 5% CO_2._ On days 3 and 5, the culture medium was supplemented with 25 ng/mL GM-CSF. On the sixth day of culture, the differentiated MDMs were used for bacterial infection.

*Growth of Mtb strains* - For the present study, the following strains were used: Mtb reference strain H37Rv and the clinical isolate strains from the EA lineage phenotype 1 (9005186), Haarlem phenotype 2 (1020319), and Haarlem phenotype 4 (1036226), all of which had distinctive restriction fragment length polymorphisms (RFLPs) and spoligotyping patterns. The strains were previously characterised as bearing different degrees of virulence in a mouse model (Marquina-Castillo et al. 2009), and were kindly donated by Dr Rogelio Hernández-Pando (National Institute of Medical Sciences and Nutrition Salvador Zubirán, Ciudad de Mexico, Mexico). Of note, these clinical isolate strains form part of a panel belonging to the clinical/epidemiological diversity of Mtb strains found in the southwest of Mexico. In a mouse model, phenotype 1 strain does not induce a protective immune response, and shows increased virulence and transmission; phenotype 2 strain induces a low anti-inflammatory cytokine response, and shows increased virulence; and phenotype 4 induces a protective adaptive immune response, and is less virulent (Marquina-Castillo et al. 2009). All strains were grown in Middlebrook 7H9 (BD-Diagnostic Systems, Sparks, MD, USA) supplemented with 10% OADC (BD-Diagnostic Systems). Strains were grown to logarithmic phase and measurement of the optical density determined the culture concentrations by spectrometry at 600 nm. Working aliquots were stored at -80ºC until their use. Bacillary viability was tested by measuring the number of colony forming units (CFU), after growth for 21 days of serial dilutions on 7H10 agar plates (BD-Diagnostic Systems) supplemented with 10% OADC (BD-Diagnostic Systems).

*MDM infection and intracellular growth of Mtb* - For infection of MDMs, a vial of the corresponding Mtb strain was thawed, and the declumping of bacterial aggregates was performed by vortexing with borosilicate beads (Sigma-Aldrich, St. Louis, MO, USA) for 5 min and centrifuged at 2040 × *g* for 5 min. MDM infection was performed at a multiplicity of infection (MOI) of 5 (i.e. a ratio of five bacteria to one macrophage). After 24 h, supernatants from infected and uninfected MDMs were harvested, treated with a protease inhibitor cocktail (Promega, Madison, WI, USA), sterilized by filtration (Sartorius Stedim Biotech, Goettingen, Germany) and stored at -80ºC until cytometric bead array (CBA) analysis. After infection, MDMs were washed with RPMI-1640 medium (Gibco, Life Technologies,) to remove non-internalised bacteria. A solution of 0.1% SDS was added to the MDMs for 10 min at room temperature to lyse the cells and, after stopping the lysis with 20% BSA, the supernatant was used to prepare serial dilutions that were plated onto Middlebrook 7H10 agar (BD-Diagnostic Systems) supplemented with 10% OADC (BD-Diagnostic Systems). CFUs were grown at 37ºC and 5% CO_2_ over a period of 21 days before counting.

*Ziehl-Neelsen staining* - Infection was performed in MDMs grown in chamber slides (Nunc Lab Tek II, Sigma-Aldrich) at an MOI of 5, over a period of 2 h at 37ºC and 5% CO_2_. During the infection, the chamber well contained RPMI-1640 medium (Gibco, Life Technologies) supplemented with 20% autologous serum. Extracellular bacteria were washed with RPMI-1640 medium and cells were fixed with 4% paraformaldehyde (PFA) for 30 min at room temperature. Ziehl-Neelsen staining was performed using the conventional protocol (Stinson et al. 2014) to identify the bacilli associated with cells. The percentage of MDMs with at least one associated bacteria was calculated after microscopic examination of the stained slides, counting at least 100 MDMs/sample using a Carl Zeiss inverted Axiovert M-200 microscope (Zeiss, Goettingen, Germany).

*Expression of cell-surface markers in Mtb-infected MDM* - MDMs infected for 24 h (or not) were recovered from 24-well plates (Corning-Costar, USA) by scratching the plate with a micropipette tip, and checking under the microscope that no cells remained attached at the plate. Cells were labelled to measure the expression of cell-surface molecules using 1:200 dilutions of the following antibodies from BD Pharmingen, Torreyana Road, San Diego, CA, USA: APC-mouse anti-human HLA-DR clone G46-6, APC-Cy7 mouse anti-human CD80 clone L307.4, PE-Cy5-mouse anti-human CD86 clone 2331 (FUN-1) and, FITC-mouse anti-human PD-L1 clone MIH1. Labelling was performed by incubating the cells for 20 min at 4ºC in the dark; cells were then washed with 1 × PBS and fixed with 4% paraformaldehyde for 30 min. Flow cytometry data was acquired using the FACS Canto II cytometer (BD Biosciences). For data analysis, FACS Diva v.6.0 (BD Biosciences) and FlowJo VX (Tristar, Ashland, OR, USA) software were used.

*Determination of cytokine and chemokine levels in supernatants of MDMs using a CBA* - The cytokines IL-8, IL-1β, IL-6, IL-10, and IL-12p70 and the chemokines CCL5/RANTES, CXCL9/MIG, and CCL2/MCP-1 were measured using CBAs (BD Bioscience), according to the manufacturer’s instructions. The limits of detection were 3.6 pg/mL for IL-8, 1 pg/mL for RANTES, 2.5 pg/mL for MIG, 2.7 pg/mL for MCP-1, 7.2 pg/mL for IL-1β, 2.5 pg/mL for IL-6, 3.3 pg/mL for IL-10, and 1.9 pg/mL for IL-12p70. Flow cytometry data was acquired using a FACS Canto II cytometer (BD Biosciences). Analysis of the data was carried using FCAP array 3.0 software (BD Biosciences).

*Statistics* - Data were compared between healthy subjects and patients with diabetes using a two-way ANOVA analysis with Bonferroni’s post-hoc test. P values ≤ 0.05 were considered significant. Comparisons within the groups (healthy subjects or patients with diabetes) were made by means of a Kruskal-Wallis test and a Dunn’s post-test assuming non-normally distributed data. Statistical analysis was performed using Graphpad Prism software v.5.0 (San Diego, CA, USA). A Spearman non-parametric correlation analysis was performed in order to exclude the interference of several variables on the outcome measurements. Partial correlations analysis (corrected for glycaemia) was performed to exclude the effect of this variable from the analysis.

## RESULTS

*Clinical and laboratory data of the participants* - An analysis of the subjects’ characteristics was performed to minimise the chances that differences may arise from differences in clinical or laboratory parameters. The comparison of the clinical parameters between patients with diabetes and healthy subjects is shown in Table. The two study groups showed similar BMI values and HDL-cholesterol and LDL-cholesterol levels, while patients with T2D showed significantly higher levels of glucose, glycated haemoglobin (HbA1c), total cholesterol, and triglycerides compared to the control group, as expected, given the inclusion criteria for the participants.

**TABLE:** Clinical and laboratory characteristics of the participants

Variable	People without diabetes (control)	Patients with type 2 diabetes	p value
Number of cases (n)	9	10	NA
Age (years)	44.78 (5.47)	56 (8.97)	0.005^*^
^a^Sex: male number (%)	1 (11.1)	2 (20)	1.000
BMI (kg/m2)	29.86 (6.32)	29.72 (2.90)	0.955
HbA1c (%)	5.64 (0.38)	11.42 (3.29)	< 0.001^***^
Glucose (mg/dl)	90.43 (13.64)	171.82 (57.00)	0.001^***^
Years with DM2 diagnosis	NA	9.7 (5.7)	---
Total cholesterol^b^ (mg/dl)	164.84 (13.22)	214.69 (47.85 )	0.021^*^
HDL-cholesterol (mg/dl)	42.19 (9.88)	43.55 (9.41)	0.769
LDL-cholesterol (mg/dl)	98.38 (13.78)	128.78 (32.18)	0.024^*^
Triglycerides^b^ (mg/dl)	121.56 (51.28)	264.94 (265.60)	0.034^*^

T tests were performed to identify differences between groups unless otherwise stated; a: Fisher test to determine if differences exist in the proportion of individuals regarding gender; b: Mann-Whitney U test were performed for these variables; mean (± standard deviation) are shown for all quantitative variables; value of alpha was set to α = 0.05; BMI: body mass index; HbA1c: glycated haemoglobin %; HDL: high density lipoprotein; LDL: low density lipoprotein; NA: non-applicable; *: p ˂ 0.05; **: p ˂ 0.01; ***: p ˂ 0.001.

Aging leads to the dysregulation of multiple components of the immune system that results in increased susceptibility to infections, and a poor response to vaccines in the aging population (immunosenescence). Dysfunction in adaptive B and T cells during aging are well documented, but the effect on innate immunity remains incompletely understood. While most immunosenescence data arise from experimental models, studies in humans have documented age effects on innate immunity in subjects ≥ 65 years (Metcalf et al. 2015). In the present work, differences in age were found between the diabetic and the non-diabetic group; for this reason, even though all the subjects evaluated were under 65 years of age, we did perform an exploratory analysis of the association of age to every variable measured to exclude any possible interference [Supplementary data ( Table)]. The analysis found that the main variable associated to the response among the variables analysed was glycaemia.

**Fig. 1:**
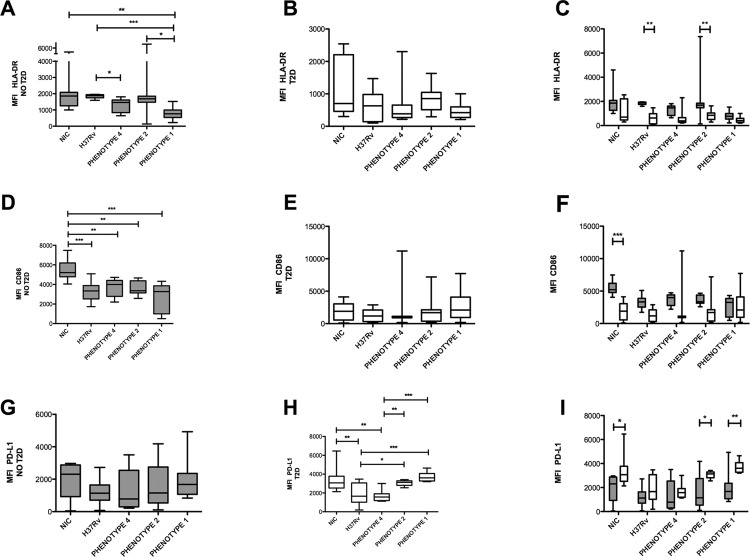
: expression of activation cell-surface markers in monocyte-derived macrophages (MDM) upon *Micobacterium tuberculosis* (Mtb) infection in the context of type 2 diabetes (T2D). Plots showing the median and interquartile range of fluorescent intensity (MFI) for MDM expression of HLA-DR, CD86, and PD-L1, as measured by flow cytometry in basal cells (NIC = non-infected control), or after Mtb infection with the indicated strains for 24 h at an MOI 5 in cells derived from healthy subjects (NO T2D, grey bars) or patients with diabetes (T2D, white bars). Intragroup comparisons were performed using a Kruskal-Wallis test followed by Dunn’s multiple comparison post-test. Panels A, D, and G show the MFI and the interquartile range for comparison in the non-diabetic group; panels B, E, and H represent the intragroup comparison for cells derived from patients with diabetes. A two-way ANOVA analysis with Bonferroni’s post-hoc test was used to compare patients with diabetes and healthy subjects following the different treatments, as shown in panels C, F, and I. For each group n = 9. *p ˂ 0.05; **p ˂ 0.01; ***p ˂ 0.001. For HLA-DR, the two-way ANOVA interaction p value was 0.3822, the diabetes status p value was < 0.0001, and the infection status p was 0.0042. For CD86, the two-way ANOVA interaction p value was 0.0368, the diabetes status p value was < 0.0001, and the infection status p value was 0.1827. For PD-L1, the two-way ANOVA interaction p value was 0.1402, the diabetes status p value was < 0.0001, and the infection status p value was < 0.0001. For CD80, the two-way ANOVA interaction p value was 0.0230, the diabetes status p value was 0.7937, and infection status p value was 0.0327.

**Fig. 2:**
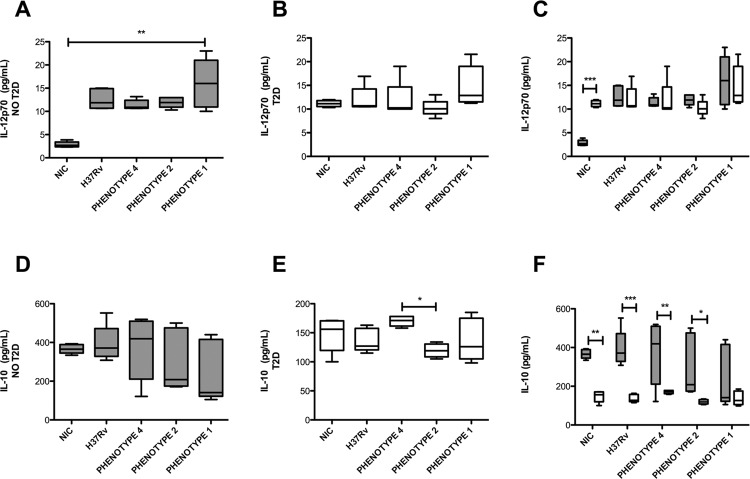
: secretion of IL-12p70 and IL-10 by monocyte-derived macrophages (MDM) from patients with diabetes and healthy subjects following *Micobacterium tuberculosis* (Mtb) infection*.* MDMs were infected over a period of 24 h at an MOI of 5, and cytokine levels in the culture supernatants were evaluated using a cytometric bead array (CBA) assay. Plots show the median and interquartile range values for basal cells (NIC = non infected control), or after Mtb infection with the indicated strains in cells derived from healthy subjects [NO type 2 diabetes (T2D), grey bars] or patients with diabetes (T2D, white bars). Intragroup comparisons were performed using a Kruskal-Wallis test and a Dunn’s multiple comparison post-test (panels A-B and D-E, respectively). A two-way ANOVA analysis with Bonferroni’s post-hoc test was used to compare the diabetic and non-diabetic groups with the different treatments, as shown in panels C and F. NO T2D group, n = 5; T2D group, n = 5; *p ˂ 0.05; **p ˂ 0.01; ***p ˂ 0.001. For IL-12p70, the two-way ANOVA interaction p value was 0.0016, the diabetes status p value was < 0.1913, and the infection status p value was < 0.0001; for IL-10, the two-way ANOVA interaction p value was 0.4874, the diabetes status p value was < 0.0001, and the infection status p value was < 0.2352.

*T2D alters MDM activation status at steady-state and after Mtb infection* - While it has been proposed that low-grade chronic inflammation is associated with T2D and tilts MFs from an anti-inflammatory phenotype towards a more pro-inflammatory one (Lumeng et al. 2007), there are only a few studies addressing how MFs from patients with T2D become activated during Mtb infection. To address this issue, monocytes derived from healthy subjects, or patients with T2D, were differentiated into MDMs, and then infected at an MOI of 5 for 24 h with the reference laboratory strain H37Rv, or three clinical isolate strains of increasing virulence (Marquina-Castillo et al. 2009) (phenotypes 4, 2, and 1, respectively). Flow cytometry analysis of the expression of surface molecules was performed in MDMs derived from both diabetic and non-diabetic subjects, as shown in Supplementary data (Fig. 1). Along with this, we also observed that even before exposure of MDMs to infection (basal level), patients with T2D exhibited lower expression levels of CD86 (an antigen-presentation molecule), and a higher level of expression of programmed death ligand 1 (PD-L1), which is a molecule that acts as a suppressor of T cell activation [Fig. 1, Supplementary data (Fig. 2)]. Upon infection with the different Mtb strains, MDMs derived from non-diabetic donors showed a decreased expression of HLA-DR (in response to the more virulent strain, phenotype 1), CD80, and CD86, whereas PD-L1 expression was not affected [Fig. 1A, D, G, Supplementary data (Fig. 2)]. In contrast, Mtb infection did not affect HLA-DR, CD80, or CD86, expression in MDMs derived from patients with T2D, and there was no effect on PD-L1 expression in response to the high virulence strains [Fig. 1B, E, H, Supplementary data (Fig. 2B)]. When the expression levels of these molecules were directly compared between patients with T2D and non-diabetic control subjects under the different infection conditions, the levels of expression of HLA-DR were significantly lower in MDM cells derived from patients with T2D infected with H37Rv or with the phenotype 2 virulent strain, compared to MDM cells derived from non-diabetic subjects infected with the same agents. PD-L1 expression was also increased in MDM cells derived from patients with T2D compared to healthy subjects, at least for the strains with the highest virulence (phenotypes 2 and 1) (Fig. 1C, F, I).

Taken together, these data show that T2D alters the expression of antigen presentation and co-stimulatory molecules in MDMs under basal conditions, and it also affects how these cells become activated following Mtb infection, as shown an by the enhanced expression of the T-cell suppressive molecule, PD-L1.

**Fig. 3:**
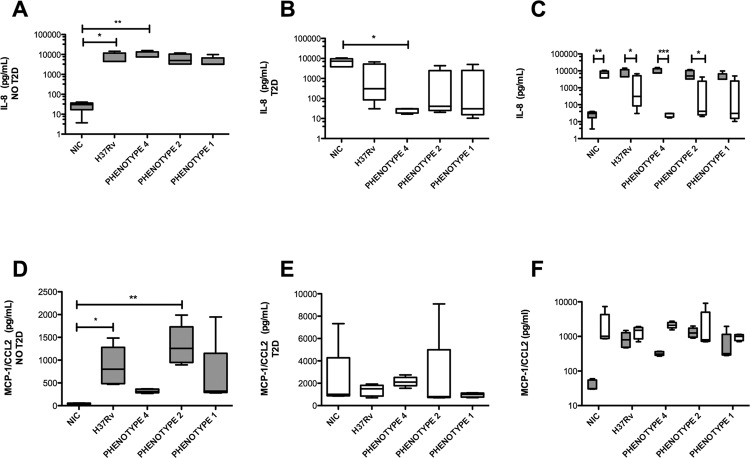
: secretion of IL-8 and MCP-1 by monocyte-derived macrophages (MDM) from diabetic and healthy subjects following *Mycobacterium tuberculosis* (Mtb) infection. MDMs from healthy subjects [NO type 2 diabetes (T2D), grey bars] and patients with diabetes (T2D, white bars) were infected over a period of 24 h at an MOI of 5. Following this, the culture supernatants were recovered and evaluated using a cytometric bead array (CBA) assays. The graphs show the median and interquartile range values of basal cells (NIC = non infected control), or after Mtb infection with the indicated strains. Intragroup comparisons were performed using a Kruskal-Wallis test and a Dunn’s multiple comparison post-test (panels A-B and D-E, respectively), while the comparison between groups was performed using a two-way ANOVA analysis with Bonferroni’s post-hoc test, as shown in panels C and F. In each study group n = 5. *p ˂ 0.05; **p ˂ 0.01; ***p ˂ 0.001. For IL-8, the two-way ANOVA interaction p value was < 0.0001, the diabetes status p value was 0.0001, and the infection status p value was 0.4187; for MCP-1, the two-way ANOVA interaction p value was 0.5764, the diabetes status p value was 0.0083, and the infection status p value was 0.6053.

*The MDM inflammatory response to Mtb is influenced by T2D* - Another feature of MF biology is how these cells respond to pathogenic challenges in terms of the production of inflammatory signals. Here, we assessed how T2D affects this process in MDMs. Under unstimulated conditions, MDMs derived from patients with T2D displayed a tendency toward higher secretion of pro-inflammatory cytokines, such as IL-12p70, IL-1, and IL-6 compared to MDMs derived from non-diabetic subjects [Fig. 2, Supplementary data (Fig. 3)]. In contrast, MDMs derived from patients with T2D cells secrete lower levels of the anti-inflammatory cytokine IL-10 compared to control MDMs (Fig. 2). Following a 24 h challenge with the different Mtb strains, MDMs derived from patients with T2DM did not show changes in cytokine secretion (Fig. 2). As expected, MDMs derived from control subjects showed a tendency to increase inflammatory signals after Mtb infection compared to non-infected cells [Fig. 2, Supplementary data (Fig. 3)].

**Fig. 4:**
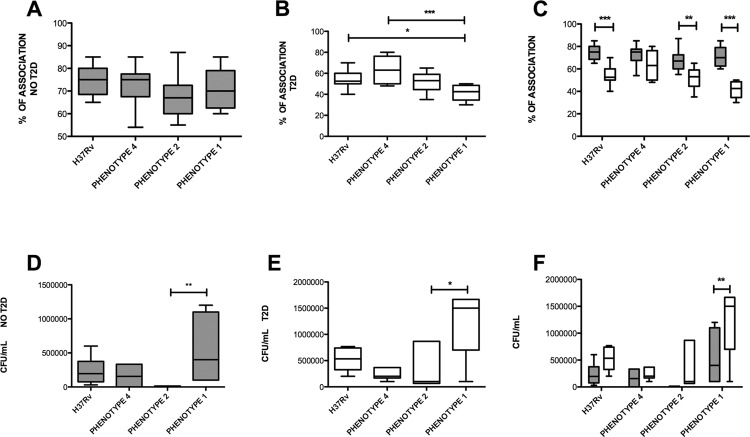
: assessment of the association (binding/intake) and control of intracellular growth of *Mycobacterium tuberculosis* (Mtb) in the context of type 2 diabetes (T2D). The percentage of binding/intake of MDMs from healthy subjects (NO T2D, panel A, grey bars) or patients with diabetes (T2D, panel B, white bars) after infection with Mtb over a 2 h period at an MOI of 5, was evaluated by Ziehl-Neelsen staining. Panel C shows a direct comparison of the percent association between the two groups of MDMs. Colony forming units (CFUs) in MDMs from healthy subjects (NO T2D, panel D, grey bars) or T2D (panel E, white bars) that were infected with the indicated strains for 2 h at a MOI of 5, and lysed after 24 h to evaluate the intracellular growth of Mtb are shown. Panel F shows a direct comparison of the CFU data between the two groups of MDMs. Data are presented as median and interquartile ranges. The intragroup comparison was performed using a Kruskal-Wallis test and a Dunn’s multiple comparison post-test (panels A-B and D-E, respectively), while the comparison between groups was performed using a two-way ANOVA analysis with Bonferroni’s post-hoc test, as shown in panels C and F. NO T2D, n = 9; T2D, n = 9. *p ˂ 0.05; **p ˂ 0.01; ***p ˂ 0.001. For bacterial association, the two-way ANOVA interaction p value was 0.0063, the diabetes status p value was < 0.0001, and the infection status p value was 0.0017. For the numbers of CFUs, the two-way ANOVA interaction p value was 0.1760, the diabetes status p value was 0.001, and the infection status p value was < 0.0001.

We next investigated the secretion of chemokines important for the recruitment of inflammatory cells to infectious sites, such as IL-8, MCP-1, MIG, and RANTES. Under unstimulated conditions, MDMs derived from patients with T2DM tended to secrete higher levels of these chemokines compared with MDMs derived from healthy subjects [Fig. 3, Supplementary data (Fig. 4)]. As expected, Mtb infection resulted in increased secretion of IL-8, MCP-1, and MIG, but it inhibited that of RANTES [Fig. 3, Supplementary data (Fig. 4)] in MDMs derived from healthy subjects. In contrast, MDMs derived from patients with T2D behaved differently upon infection, as they sustained a high production of IL-8, MCP-1, and RANTES [Fig. 3, Supplementary data (Fig. 4)]. In the case of MIG, MDMs derived from patients with T2D exhibited different secretion patterns depending on the virulence of the Mtb clinical isolates, that is, those strains with a higher virulence (phenotypes 1 and 2) provoked higher secretion compared to those with a lower virulence (phenotype 4) [Supplementary data (Fig. 4B)].

Collectively, MDMs derived from patients with T2D exhibited altered basal levels of production of inflammatory signals and showed an altered potential to respond adequately to an Mtb challenge.

*T2D diminishes the capacity of MDMs to bind, internalise, and control Mtb infection* - Previous studies have shown that MDMs derived from patients with T2D show decreased Mtb binding/intake and killing ability by both human monocytes and murine MFs (Restrepo et al. 2014), but until now there has been no evidence to suggest whether it alters these features in human MFs. To address this issue, we assessed the effects on Mtb bacterial association with MDMs derived from patients with T2D, comparing the Mtb reference strain with the clinical isolates having different levels of virulence. Ziehl-Neelsen staining was used to identify acid-fast bacilli associated with the MDMs, and to quantify the percentage of MDMs associated with at least one bacterium. As shown in Fig. 4A, the virulent reference strain (H37Rv), and the strains bearing different degrees of virulence, had the same levels of association with MDMs derived from non-diabetic subjects. MDMs derived from patients with T2D exhibited a similar level of association for all Mtb strains, with the exception of the hyper virulent strain (phenotype 1) of the LAM lineage whose level of association with the aforementioned MDMs was significantly reduced (Fig. 4B) compared to the reference strain and to less virulent strains. When the levels of bacterial association were directly compared between MDMs derived from patients with T2D or control donors, T2D cells displayed an impaired capacity to associate with the more virulent Mtb strains compared to control cells (Fig. 4C). With respect to the capacity to control intracellular bacterial growth, MDMs derived from patients with T2D were more permissive for the highly virulent Mtb strain phenotype 1, as compared to MDMs derived from non-diabetic control subjects (Fig. 4D-F).

Taken together, these data demonstrate that, even though T2D impairs association (binding and intake) of the most virulent Mtb strains, this metabolic disease also renders MFs more permissive to intracellular Mtb growth.

## DISCUSSION

Little information is available on the molecular epidemiology in Mexico of *Mycobacterium* species. However, the published data report a variety of lineages that are prevalent in the surveyed regions (Lopez-Rocha et al. 2013, Vera-Cabrera et al. 2014, Flores-Treviño et al. 2015, Zenteno-Cuevas et al. 2015). While it is clear that T2D constitutes a risk for the development of TB, the immunological abnormalities that contribute to this event and the influence of the virulence of Mtb strains are less clear. In the present study, we provide evidence suggesting that the basal phenotype and inflammatory potential of MFs is altered in patients with T2D. Moreover, these cells appear to exhibit a deficient capacity to associate (bind and internalise), respond, and control the bacillus, particularly against the more virulent Mtb strain among the three clinical isolates previously characterised by their distinctive epidemiological and immunological profiles (Marquina-Castillo et al. 2009). All things considered, while preliminary in its nature, mainly due to the small sample size of analysed subjects, we estimate this study makes three major contributions to our understanding of the co-morbidity established between T2D and TB.

First, we show that T2D alters the MF activation status under basal conditions and following Mtb infection. As the basal expression of molecules important in macrophage activation has not been previously reported in patients with diabetes, we evaluated the expression of HLA-DR, CD80, CD86, and PD-L1 in MDMs derived from patients with diabetes compared to that in MDMs derived from healthy subjects. Our results showed T2D modifies the MDM activation status by decreasing the basal expression levels of HLA-DR, CD80, and CD86, mirrored by an increase in PD-L1 expression. These data are consistent with a recent report in which treatment with high glucose and palmitate synergised to inhibit HLA-DR and CD86 expression in the human monocyte cell line THP1 (Xiu et al. 2016). With the exception of PD-L1, MDM cells from patients with T2D failed to modulate the expression of the activation associated molecules in response to Mtb infection, as compared to MDMs from healthy subjects, suggesting that the MDM-derived T2D cells are unresponsive to pathogenic challenge. Perhaps the most interesting result comes from the direct comparison between healthy subjects and patients with T2D with regards to PD-L1 expression, which remained high in T2D cells under both basal levels and after infection with the more virulent Mtb strain. This is an important finding because an increased PD-L1 expression in mononuclear phagocytes is associated with the inhibition of T cell proliferation and IFN-g in the context of TB, the PD-1/PD-L1 pathway inhibits T cell effector functions during TB, and a high PD-L1/CD86 ratio is part of the CD16^+^/CD163^+^/MerTK^+^/PD-L1/pSTAT3^+^ signature in MFs that are susceptible to Mtb infection, and possess immunosuppressive activity toward the Th1 response (Lastrucci et al. 2015). The characterisation of phenotypic markers of MF activation during T2D has not been reported, but it is probably influenced by metabolites as activators (*e.g.*, palmitate), as has recently been demonstrated in the context of obesity (Kratz et al. 2014). We predict that T2D promotes a unique program of MF activation during TB with a deleterious effect on the ability of MFs to present antigens and activate a Th1 response against Mtb, representing an issue that we will address in the future.

Second, we show that the MF inflammatory response to Mtb is influenced by T2D. At the cytokine level, MDMs derived from patients with T2D displayed a tendency towards higher secretion of pro-inflammatory signals under basal conditions (IL-12p70, IL-1-b, and IL-6), whereas the anti-inflammatory signal IL-10 was significantly downregulated in T2D MFs. The pro-inflammatory environment in T2D is characterised by elevated cytokine and acute phase reactant levels, increased markers of leukocyte activation, and increased MF infiltration into adipose and other tissues. In particular, adipose tissue-related production of pro-inflammatory molecules contributes to a low grade systemic inflammation seen in chronic diseases associated with the metabolic syndrome (Lumeng & Saltiel 2011). Therefore, baseline MFs may be prone to contributing to the pro-inflammatory vicious cycle, characteristic of patients with T2D. However, the MDMs derived from patients with T2D were unresponsive towards all Mtb strains. This is in line with a recent report describing the deficient production of TNF-α, IL-6, and MCP-1 in response to different *Mycobacterium* species by MFs isolated from mice with diabetes (Alim et al. 2017). Moreover, the marked reduction in the production of IL-10 may be of consequence, because this cytokine plays a protective role in T2D and in the prevention of immunopathology induced by TB. In the context of T2D, for example, a study has shown that MFs cultured under physiologically relevant hyperglycaemic conditions display hypo responsiveness to IL-10, resulting in a failure to down-regulate TNF-α production, implying that a reduction of IL-10 activity in MFs may contribute to the chronic low-grade inflammation seen in T2D (Barry et al. 2016). In the context of TB, IL-10 plays a role in the balance between the protective immune response to Mtb infection and immunoregulatory mechanisms to prevent tissue damage. Reciprocally, Mtb hijacks the production of this cytokine to increase its fitness in the host (Redford et al. 2011). Therefore, while it might be tempting to propose that the drop in production of IL-10 by T2D MFs may enhance the microbicidal effect against Mtb, it is also probable that the pro-inflammatory nature of the cells may contribute to tissue damage and immunopathology due to TB. The latter option was indeed the case in diabetic mice infected with *Mycobacterium fortuitum*, which displayed a higher number of inflammatory foci and percent area of inflammation in the liver (although the lung and spleen were not analysed) (Alim et al. 2017).

As with cytokines, MDMs derived from patients with T2D display a tendency to secrete higher levels of chemokines, such as IL-8, MCP-1, MIG, and RANTES under basal conditions, as compared to MDMs from control subjects. To our knowledge, this is the first time that the levels of MCP-1, RANTES, and MIG have been reported in MDMs derived from patients with diabetes. High glucose treatment increases MCP-1 expression/release in several cell types, including THP-1 cells (Stew et al. 2013). This is of importance, given that the MCP-1/CCR2 axis plays a central role during the promotion of adipose tissue MF recruitment and insulin resistance (Xu et al. 2015). In addition, baseline monocytes in patients with T2D with TB have been reported to express higher levels of the MCP-1 receptor (CCR2) (Stew et al. 2013). With respect to RANTES, its receptor CCR5 has been reported to be highly overexpressed in PBMCs derived from patients with T2D (Dytfeld et al. 2006), suggesting that T2D MFs may be prone to recruiting circulating leukocytes into the tissues they are resident in. As expected, in the context of Mtb infection, MDMs derived from healthy subjects displayed increased secretion of IL-8, MCP-1, and MIG. In contrast, MDMs derived from patients with T2D showed a down-regulation of IL-8 secretion despite the sustained production of MCP-1 and RANTES. The case of IL-8 is quite interesting since IL-8 is involved in the recruitment of neutrophils and other granulocytes to the mycobacteria-infected lung (Slight & Khader 2013). In fact, this cytokine is mainly responsible for the interaction between neutrophils and MFs in infectious disease. Based on the aberrant high baseline levels of IL-8, we predict that T2D induces a frequent but unhealthy cooperation between these cells contributing to chronic low-grade inflammation; in the presence of the bacillus, however, this cooperation is disrupted due to the shutdown of IL-8 production, as observed in MFs derived from patients with T2D, leading to the consequent potential arrest of neutrophil influx. As neutrophils directly influence the development of an adaptive immune response, their failure to be recruited by T2D MFs during infection may result in a defective Th1 response against Mtb. Unlike other chemokines, we observed that T2D MFs secrete higher levels of MIG based on the degree of virulence of the Mtb clinical isolates, compared to control MFs. While there are no reports on MIG production by MFs derived from patients with T2D, MIG is still considered to be a promising biomarker candidate for TB diagnosis and treatment monitoring (Jacobs et al. 2016), further supporting the concept that T2D dysregulates the MF inflammatory response disproportionally against virulent Mtb strains. Future studies will include an assessment of the signalling pathways responsible for such discrepancies between MFs derived from patients with T2D compared to those from healthy subjects, and measurement of the chemoattractant potential of supernatants derived from Mtb-infected T2D MFs to induce the migration of neutrophils, among others.

The third, and last, contribution of this study is the demonstration that T2D diminishes the capacity of MDMs to bind, internalise, and control virulent Mtb strains. Indeed, in direct comparison with control cells, MDMs derived from patients with T2D displayed an impaired capacity to associate with the more virulent Mtb strain. This is an interesting result since these features may be affected by intrinsic alterations associated with diabetes and/or by differences in the cell wall composition of Mtb strains. Concerning the potential alterations in host cells, hyperglycaemia results in protein glycation and the formation of advanced glycation end products (AGEs), which can cause an impairment of host proteins involved in complement activation, bacterial uptake, phagocytic killing, scavenging of bio limiting nutrients, and changes in the binding of host surface receptors for pathogens (Gan 2013). In particular, highly glycated proteins could bind to molecules that are very important for mycobacterial phagocytosis, such as antibodies, complement, the mannose receptor, Toll-like receptors, and scavenger receptors. In addition, the fact that high concentrations of glucose disrupt C-type lectin function by mechanisms involving competitive inhibition of carbohydrate-binding, it might contribute to poor recognition of bacterial components, and therefore to decreased phagocytosis. In agreement with our results, monocytes from patients with T2D with poor glycaemic control showed a reduced ability to associate with Mtb H37Rv, an effect attributed at least in part to alterations in complement receptors (CR3) or Fc-*γ* receptors (Fc*γ*Rs) (Restrepo et al. 2014). With regards to the effects induced by mycobacterial cell wall composition, several studies have pointed out that cell wall components have an impact on the phagocytic process (Torrelles & Schlesinger 2010). The surface of Mtb is particularly rich in mannose-containing biomolecules, including mannose-capped lipoarabinomannan (ManLAM), lipomannan (LM), PIMs, arabinomannan, mannan, and manno-glycoproteins. PIMs, LM, and ManLAM are exposed on the Mtb cell surface, and they act as ligands for host cell receptors and contribute to the pathogenesis of Mtb (Torrelles & Schlesinger 2010). Future studies will analyse the cell wall composition of the Mtb strains tested in this study and determine whether the amount and nature of mannose groups exposed on the surface of these strains account for the difference in bacterial association rate observed in MDMs derived from patients with T2D. Likewise, an extensive phenotypic analysis of the phagocytosis receptor repertoire will be conducted in MDMs derived from patients with T2D; we predict that the expression of the phagocytosis receptors is downregulated compared to that in control MDMs.

In spite of the lower bacterial association, reflecting reduced bacterial binding/intake rates compared to control cells, MDMs from patients with T2D displayed a higher intracellular load of the hyper virulent clinical isolate phenotype 1. Based on this, we predict that the T2D environment limits the MF microbicidal response against Mtb. Previous reports have shown that leukocytes from patients with diabetes exhibit a defective response to oxidative stress. In fact, neutrophils from patients with T2D display reduced O_2_- production and correlated with an impairment of bactericidal capacity. Similarly, a decreased ratio of reduced (GSH) to oxidised (GSSG) glutathione is indicative of oxidative stress, and this major redox regulator is affected in diabetes. This was shown by the decreased GSH ratio in patients with T2D with poor glycaemic control due to the compromised levels of GSH synthesis and metabolism enzymes (Lagman et al. 2015). In addition, both medium containing high glucose, and hyperglycaemic sera derived from patients with T2D, reduced the *in vitro* differentiation of monocytes into functional DCs, which exhibit defective activation by PAMPs or DAMPs (Montani et al. 2016). Finally, a study from our group found that, in patients with T2D with latent or active TB, there is a lower expression of antimicrobial peptides (cathelicidin-LL37, human neutrophil peptide-1, and human beta-defensins-2 and -3), compared to healthy subjects (González-Curiel et al. 2011). All this evidence is supported by a recent report demonstrating that diabetic mice are susceptible to *M. fortuitum* infection, displaying a high bacterial load in lung, liver, and spleen (Alim et al. 2017). Noticeably, alveolar and intraperitoneal MFs from diabetic mice exhibited a lower capacity to phagocytose mycolic acid-coated beads, as well as *M. fortuitum*, and diminished the killing activity against *M. fortuitum* as compared to control MFs (Alim et al. 2017).

As mentioned previously, the conclusions drawn from our study are limited by several potential biases. Among these are the small sample size used in our study, and the limited amount of peripheral blood available from each patient (for ethical reasons), which precludes performing several analysis in the same biological sample. Therefore, our results should be interpreted as preliminary. Indeed, if both parameters could have been increased, we would have been able to conduct a control for cofounders such as age, which was noticeably higher in the T2D group, as well as for sex, as most of the subjects included were females. Another technical limitation in this study is the lack of an evaluation of cell viability, which was not tested during our flow cytometry analyses, which is problematic given that the MOI used for MDM infection could induce apoptosis, even at the very short infection time used (24 h) after which the expression of all markers was evaluated. Finally, since the susceptibility of patients with diabetes to TB has been principally documented in individuals with bad hyperglycaemic control, we included only subjects with high HbA1c values and, as expected, those patients also displayed other metabolic features such as dyslipidaemia. Therefore, our data cannot be generalised to patients with tight glycaemic control, and/or exhibiting normal levels of cholesterol and triglycerides.

*In conclusion* - All things considered, our study provides evidence suggesting for the first time that MFs (derived from monocytes) from patients with T2D have an altered response against hyper virulent Mtb strains in terms of activation markers (important for antigen presentation), production of inflammatory signals (crucial for TB aetiology), and association with bacilli and subsequent microbicidal activity. As mentioned previously, further studies are needed to precisely determine what signals within the low-grade chronic inflammation in T2D are responsible for altering the monocyte to MF differentiation, what signalling pathways are affected within T2D MFs to decrease their microbicidal capacity, and whether these defects could be reversed to reduce susceptibility to TB. Another essential question is whether the T2D effect in MFs alters the formation and maintenance of TB granulomas. The answers to these questions may help to improve TB control in at-risk populations, such as patients with T2D.
