# Supplementary material for: Type-2 diabetes alters the basal phenotype of human macrophages and diminishes their capacity to respond, internalise, and control Mycobacterium tuberculosis
Source: Mem Inst Oswaldo Cruz. 2018 Feb 19;113(4):e170326. doi: 10.1590/0074-02760170326 (PMC5851047; doi:10.1590/0074-02760170326)
Supplement: Supplementary file 1 [file 0074-0276-mioc-113-4-e170326-Suppl01.pdf]

TABLE

Univariate correlation analysis and Glycaemia corrected partial correlation analysis of age with all analysed variables

| | Uncorrected correlation Age vs any variable in column 1 | | Glycaemia corrected partial correlations Age vs any variable in column 1 | |
|---|---|---|---|---|
| | Rho value | p value | Rho value | p value |
| Glucose (mg/dL) | 0.596** | 0.007 | NA | NA |
| Hemoglobin (percent) | 0.548* | 0.023 | 0.565 | 0.618 |
| DM2 Years of evolution | 0.488 | 0.153 | 0.948 | 0.206 |
| Weight (Kg) | -0.005 | 0.985 | 0.935 | 0.231 |
| Height (m) | -0.305 | 0.269 | 0.431 | 0.717 |
| Body Mass Index | 0.055 | 0.852 | 0.777 | 0.433 |
| Total Cholesterol_(mg/dl) | 0.145 | 0.567 | -0.943 | 0.216 |
| Triglycerides (mg/dl | 0.157 | 0.533 | -0.811 | 0.398 |
| HDL(mg/dl) | 0.062 | 0.807 | 0.174 | 0.889 |
| LDL(mg/dl) | 0.012 | 0.961 | -0.901 | 0.286 |
| HLA_DR_NIC | -0.409 | 0.092 | -0.996 | 0.058 |
| HLA_DR_H37Rv | -0.610** | 0.007 | 0.996 | 0.058 |
| HLA_DR_PHE4 | -0.412 | 0.090 | 0.842 | 0.363 |
| HLA_DR_PHE2 | -0.488* | 0.040 | 0.984 | 0.113 |
| HLA_DR_PHE1 | -0.186 | 0.459 | 0.696 | 0.510 |
| CD80_NIC | -0.251 | 0.315 | -0.969 | 0.158 |
| CD80_H37Rv | -0.317 | 0.200 | 0.099 | 0.937 |
| CD80_Phe4 | -0.309 | 0.212 | 0.308 | 0.800 |
| CD80_Phe2 | -0.065 | 0.797 | 0.226 | 0.855 |
| CD80_Phe1 | 0.331 | 0.180 | 0.513 | 0.657 |
| CD86_NIC | -0.741** | 0.000 | -0.865 | 0.334 |
| CD86_H37Rv | -0.390 | 0.099 | -0.274 | 0.823 |
| CD86_Phe4 | -0.233 | 0.337 | 0..999 | 0.029 |
| CD86_Phe2 | -0.290 | 0.228 | 0.546 | 0.633 |
| CD86_Phe1 | 0.049 | 0.841 | -0.054 | 0.966 |
| PD-L1_NIC | 0.540* | 0.017 | -0.983 | 0.119 |
| PD-L1_H37Rv | 0.327 | 0.172 | -1.000 | 0.017 |
| PD-L1_Phe4 | -0.106 | 0.665 | 0.505 | 0.663 |
| PD-L1_Phe2 | 0.131 | 0.593 | -0.475 | 0.685 |
| PD-L1_Phe1 | 0.515* | 0.024 | -0.999 | 0.032 |
| IL-1B_NIC | 0.512 | 0.130 | -0.917 | 0.262 |
| IL-1B_H37Rv | 0.310 | 0.384 | -0.754 | 0.456 |
| IL-1B_Phe4 | 0.560 | 0.093 | -0.982 | 0.120 |
| IL-1B_Phe2 | 0.719* | 0.019 | -0.343 | 0.777 |
| IL-1B_Phe1 | 0.452 | 0.189 | -0.769 | 0.442 |
| IL_6_NIC | 0.659* | 0.038 | -0.428 | 0.675 |
| IL_6_H37Rv | 0.280 | 0.432 | -0.901 | 0.286 |
| IL_6_Phe4 | 0.498 | 0.143 | -0.488 | 0.675 |
| IL_6_Phe2 | 0.471 | 0.169 | -0.901 | 0.286 |
| IL_6_Phe1 | 0.055 | 0.880 | -0.923 | 0.251 |
| IL-8_NIC | 0.613 | 0.059 | -0.383 | 0.750 |
| IL-8_H37Rv | -0.710* | 0.021 | -0.623 | 0.572 |
| IL-8_Phe4 | -0.722* | 0.018 | 0.407 | 0.733 |
| IL-8_Phe2 | -0.584 | 0.076 | -0.946 | 0.210 |
| IL-8_Phe1 | -0.753* | 0.012 | -0.996 | 0.057 |
| IL-12p70_NIC | 0.805** | 0.005 | -0.027 | 0.983 |
| IL-12p70_H37Rv | -0.476 | 0.165 | -0.994 | 0.072 |
| IL-12p70_Phe 4 | -0.254 | 0.479 | 0.644 | 0.555 |
| IL-12p70_Phe2 | -0.709* | 0.022 | -0.831 | 0.376 |

| | Uncorrected correlation Age vs any variable in column 1 | | Glycaemia corrected partial correlations Age vs any variable in column 1 | |
|---|---|---|---|---|
| | Rho value | p value | Rho value | p value |
| IL-12p70_Phe1 | -0.061 | 0.867 | 0.343 | 0.777 |
| IL-10_NIC | -0.774* | 0.009 | 0.990 | 0.089 |
| IL-10_H37Rv | -0.854** | 0.002 | 0.620 | 0.574 |
| IL-10_Phe4 | -0.343 | 0.333 | -0.312 | 0.798 |
| IL-10_Phe2 | -0.848** | 0.002 | -0.548 | 0.631 |
| IL-10_Phe1 | -0.482 | 0.159 | -0.973 | 0.149 |
| MCP-1_NIC | 0.756* | 0.011 | -1.000 | 0.003 |
| MCP-1_H37Rv | 0.451 | 0.191 | 0.156 | 0.900 |
| MCP-1_Phe4 | 0.787** | 0.007 | 0.209 | 0.866 |
| MCP-1_Phe2 | -0.323 | 0.362 | -0.995 | 0.066 |
| MCP-1_Phe1 | 0.329 | 0.353 | 0.664 | 0.538 |
| MIG_NIC | 0.694* | 0.026 | 0.388 | 0.746 |
| MIG_H37Rv | -0.346 | 0.328 | 0.652 | 0.548 |
| MIG_Phe4 | -0.313 | 0.379 | 0.941 | 0.219 |
| MIG_Phe2 | 0.722* | 0.018 | -0.846 | 0.358 |
| MIG_Phe1 | 0.731* | 0.016 | 0.995 | 0.061 |
| IP-10_NIC | -0.349 | 0.324 | -0.958 | 0.184 |
| IP-10_H37Rv | -0.313 | 0.379 | 0.031 | 0.981 |
| IP-10_Phe4 | -0.835 | 0.003 | -0.998 | 0.045 |
| IP-10_Phe2 | 0.560 | 0.093 | -1.000 | 0.010 |
| IP-10_Phe1 | -0.299 | 0.402 | 0.353 | 0.771 |
| RANTES_NIC | 0.049 | 0.894 | -0.614 | 0.579 |
| RANTES_H37Rv | -0.738* | 0.015 | 0.108 | 0.931 |
| RANTES_Phe4 | 0.547 | 0.101 | 0.236 | 0.848 |
| RANTES_Phe2 | 0.640* | 0.046 | -0.576 | 0.609 |
| RANTES_Phe1 | 0.439 | 0.204 | 0.336 | 0.782 |
| Perc_FAGOCITOSIS_H37Rv | -0.455 | 0.058 | -0.070 | 0.955 |
| Perc_FAGOCITOSIS_Phe4 | -0.244 | 0.328 | -0.637 | 0.560 |
| Perc_FAGOCITOSIS_Phe2 | -0.348 | 0.157 | -0.973 | 0.147 |
| Perc_FAGOCITOSIS_Phe1 | -0.607** | 0.008 | -0.924 | 0.249 |

*: $p < 0.05$; **: $p < 0.01$.

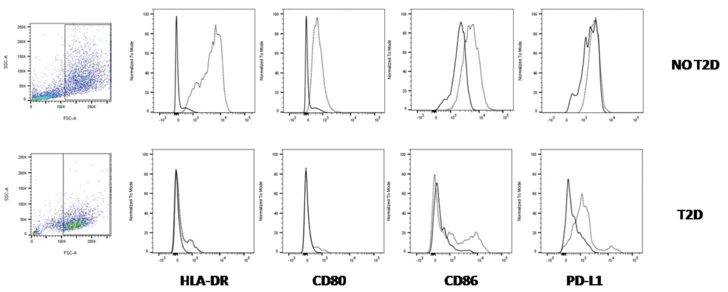


Fig. 1: flow cytometry gating strategy of a representative sample of monocyte-derived macrophages (MDM) in control healthy subjects (NO T2D) and T2D groups. Expression of the activation molecules HLA-DR, CD80, CD86, and PD-L1 in uninfected MDMs from the NO T2D and T2D groups. The solid line represents the negative control (without fluorescence mark) and the dotted line shows the study groups. To define the gate for MDM, we first established the gate for monocytes: CD14 cells were purified by negative selection of peripheral blood mononuclear cells (PBMCs), and cell dispersion was observed based on side and forward scatter parameters, following this CD14+/CD3- cells were identified (using anti-CD3-PE and anti-CD14-FITC labelled antibodies) and back gated to the side and forward scatter plot. Based on the back gating parameters, a gate for monocytes was then established and later used to identify MDMs, as shown here.

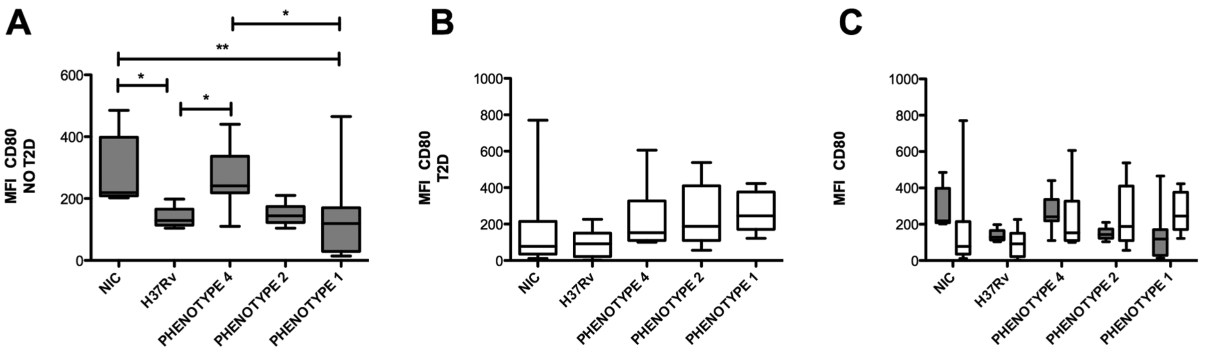


Fig. 2: expression of costimulatory cell-surface markers in monocyte-derived macrophages (MDM) following *Mycobacterium tuberculosis* (Mtb) infection in the context of type 2 diabetes (T2D). Plots showing the median and interquartile range of fluorescent intensity (MFI) for MDM expression of CD80, as measured by flow cytometry in basal cells (NIC = non infected control), or after Mtb infection with the indicated strains for 24 h at an multiplicity of infection (MOI) of 5 in cells derived from healthy subjects (NO T2D, grey bars) or patients with diabetes (T2D, white bars). Intragroup comparisons were performed with a Kruskal-Wallis test followed by Dunn's multiple comparison post-test. Panel A, show the MFI and interquartile range for the comparison in the healthy control group; panel B represent the intragroup comparison for cells derived from patients with diabetes. A two-way ANOVA analysis with Bonferroni's post-hoc test was used to compare patients with diabetes and healthy subjects subjected to different treatments, as shown in panel C. For each group n = 9. *p < 0.05; **p < 0.01; ***p < 0.001.

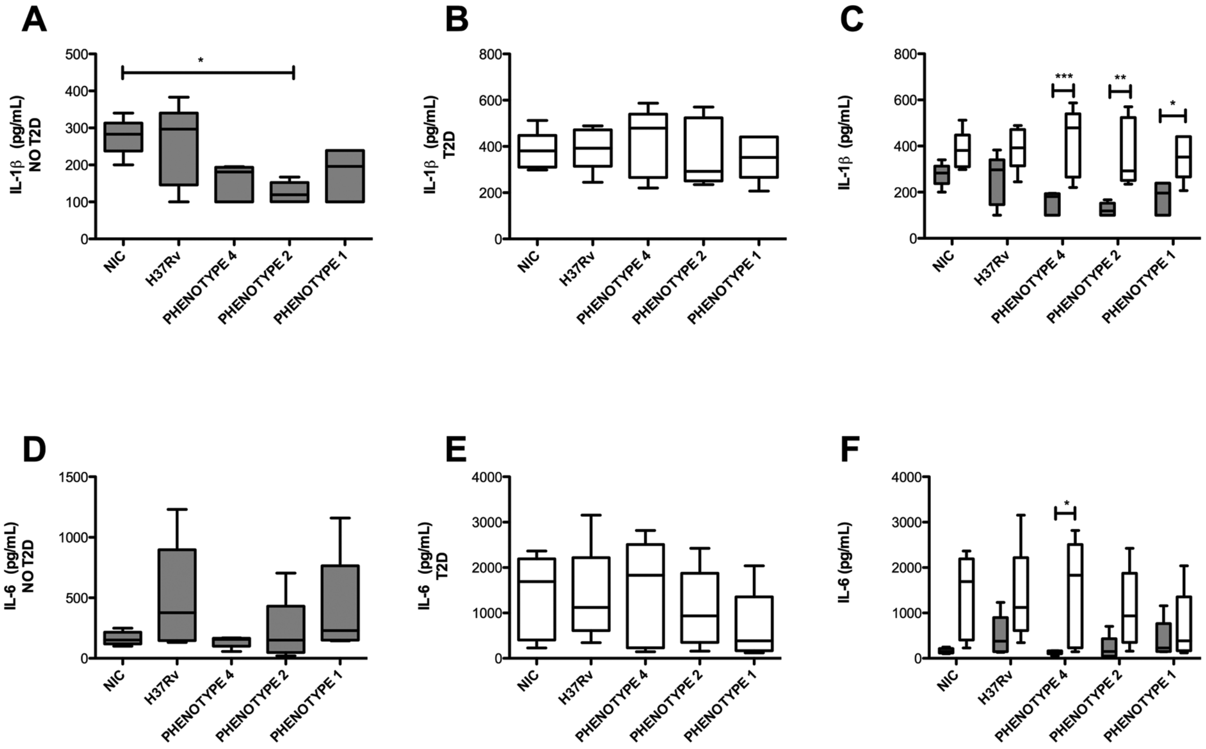


Fig. 3: secretion of IL-1β and IL-6 by monocyte-derived macrophages (MDM) from patients with diabetes and healthy subjects upon *Mycobacterium tuberculosis* (Mtb) infection. MDMs were infected over a period of 24 h at an multiplicity of infection (MOI) of 5, and the cytokine levels in the culture supernatants were evaluated using a cytometric bead array (CBA) assay. Plots show the median and interquartile range values in basal cells (NIC = non infected control), or after Mtb infection with the indicated strains in cells derived from healthy subjects [NO type 2 diabetes (T2D), grey bars] or patients with diabetes (T2D, white bars). Intragroup comparisons were performed using a Kruskal-Wallis test and Dunn's multiple comparison post-test (panels A-B and D-E, respectively). A two-way ANOVA analysis with Bonferroni's post-hoc test was used to compare patients with diabetes and healthy subject groups subjected to different treatments, as shown in panels C and F. NO T2D group, n = 5; T2D group, n = 5; *p < 0.05; **p < 0.01; ***p < 0.001. For IL-1b the two-way ANOVA interaction P value was 0.2941, the diabetes status p value was < 0.0001, and the infection status p value was 0.2498; for IL-6, the two-way ANOVA interaction p value was 0.5300, the diabetes status p value < 0.0001, and the infection status p value was 0.8092.

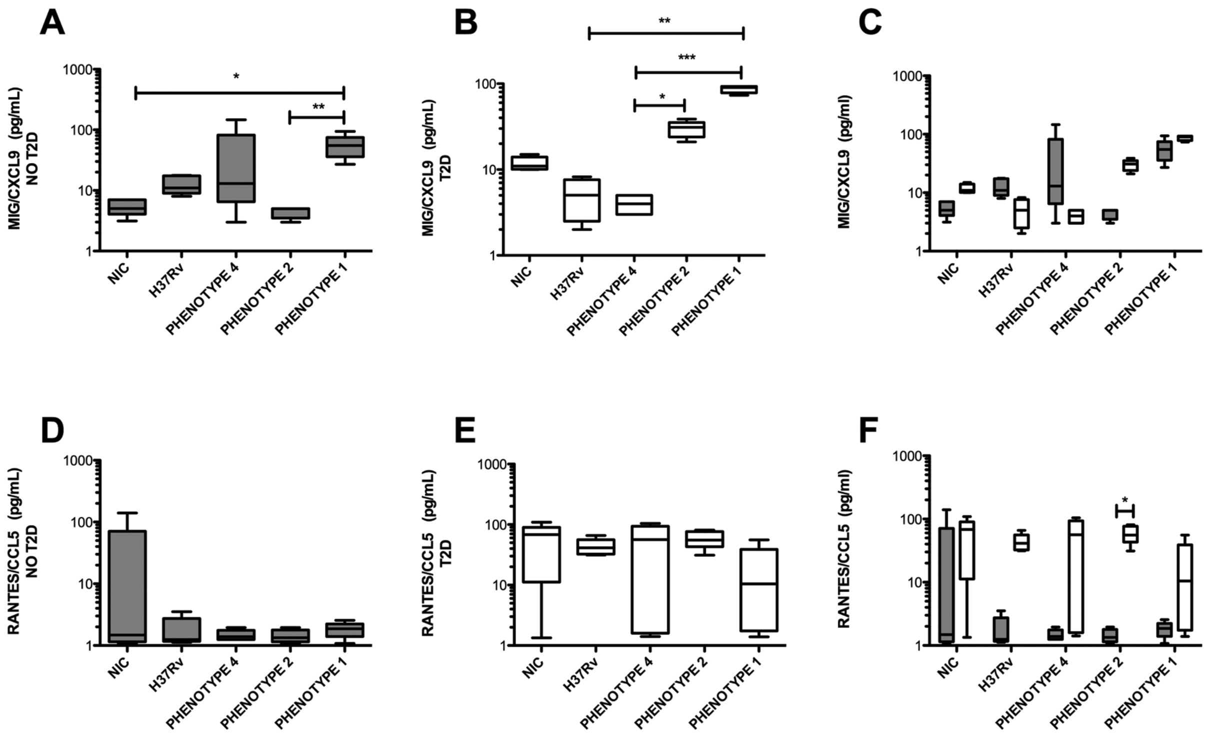


Fig. 4: secretion of MIG and RANTES by monocyte-derived macrophages (MDM) from patients with diabetes and healthy subjects following *Mycobacterium tuberculosis* (Mtb) infection. MDMs from healthy subjects [NO type 2 diabetes (T2D), grey bars] and patients with diabetes (T2D, white bars) were infected over a period of 24 h at an multiplicity of infection (MOI) of 5, culture supernatants were recovered and evaluated using a cytometric bead array (CBA) assay. Graphs show the median and interquartile range values in basal cells (NIC = non infected control), or after Mtb infection with the indicated strains. Intragroup comparisons were performed using a Kruskal-Wallis test and Dunn's multiple comparison post-test (panels A-B and D-E, respectively), while comparison between groups was performed by a two-way ANOVA analysis with Bonferroni's post-hoc test, as shown in panels C and F. In each study group n = 5. *p < 0.05; **p < 0.01; ***p < 0.001. For MIG, the two-way ANOVA interaction p value was 0089, the diabetes status p value was 0.4765, and the infection status p value was < 0.0001; for RANTES, the two-way ANOVA interaction p value was 0.5400, the diabetes status p value was < 0.0001, and the infection status p value was 0.2297.
